# Larval performance of *Zophobas morio* (F.) (Coleoptera: Tenebrionidae) on various diets enriched with post-distillation residues and essential oils of aromatic and medicinal plants

**DOI:** 10.1007/s11356-024-32603-8

**Published:** 2024-04-02

**Authors:** Marina Gourgouta, Stefanos S. Andreadis, Eleni I. Koutsogeorgiou, Christos I. Rumbos, Katerina Grigoriadou, Ilias Giannenas, Eleftherios Bonos, Ioannis Skoufos, Christos G. Athanassiou

**Affiliations:** 1 Laboratory of Entomology and Agricultural Zoology, Department of Agriculture, Crop Production and Rural Environment, University of Thessaly, Phytokou str, 38446 N. Ionia, Magnesia Greece; 2 Institute of Plant Breeding and Genetic Resources, Hellenic Agricultural Organization (DIMITRA), 57001 Thermi, Greece; 3 Laboratory of Nutrition, School of Veterinary Medicine, Aristotle University of Thessaloniki, Thessaloniki, Greece; 4 Laboratory of Animal Production, Nutrition and Biotechnology, University of Ioannina, Arta, Greece

**Keywords:** Edible insects, Waste management, Larval development, Feed conversion efficiency, Essential oils, Plant-based residues

## Abstract

The increasing demands for resources driven by the global population necessitate exploring sustainable alternatives for affordable animal protein over the use of traditional protein sources. Insects, with their high protein content, offer a promising solution, especially when reared on agricultural post-distillation residues for enhanced sustainability and cost-effectiveness. We assessed the development of *Zophobas morio* (F.) (Coleoptera: Tenebrionidae) larvae on diets enriched with essential oils and post-distillation residues from Greek aromatic and medicinal plants. Two aromatic plant mixtures (A and B) were examined. Mixture A consisted of post-distillation residues, while Mixture B incorporated these residues along with essential oils. Insect rearing diets were enriched with different proportions (10, 20, and 30 %) of these mixtures, with wheat bran serving as the control. Enrichment positively influenced larval development without compromising survival. Larval weight remained unchanged with Mixture A, but improved with Mixture B. No adverse effects were detected in the case of the enriched diets, although higher concentrations of Mixture B prolonged development time.

**Supplementary Information:**

The online version contains supplementary material available at 10.1007/s11356-024-32603-8.

## Introduction

The continuously increasing global population has resulted in a significant surge in the demand for resources (Boland et al. 2013; Searchinger et al. 2018; FAO 2019). According to the Food and Agricultural Organization (2019), by 2050, world population is estimated to approach nearly 10 billion. In addition, upward mobility and urbanization of developing countries have led to high global food demand and animal protein consumption (Teguia and Beynen 2005; Msangi and Rosegrant 2011; Van Huis and Gasco 2023; Li 2023). By 2027, it is projected that the per capita meat consumption worldwide will rise by more than 1 kg in retail weight equivalent (OECD-FAO 2018). Moreover, the rise in meat consumption will be even more pronounced in developing nations, where the per capita consumption of animal protein is estimated to increase by 22% and 25% by 2030 and 2050 respectively (FAO/WHO 2017). In recent years, the world market prices of several agricultural commodities have escalated at a staggering pace (Zhao et al. 2022; Li 2023). The rise in world prices for major agricultural crops will result in an increase of over 30% in the prices of beef, pork, and poultry by 2050 compared to the prices of 2000 (Nelson et al. 2009). Thus, it has become imperative to explore and identify alternative sources that can contribute to a sustainable and cost-effective supply which will increase the availability of both animal protein and grains, while keeping their prices low.

The use of insects as a source of food and feed holds great promise, given their high protein content and nutritional value as long as the fact that their production is sustainable and aligns with modern circular economy practices (Van Huis and Oonincx 2017; Koutsos et al. 2019; Rumbos et al. 2022). It is worth noting that the consumption of insects is a natural dietary practice for several species, such as pigs, poultry, and fish (Veldkamp et al. 2012; Makkar et al. 2014; Henry et al. 2015). Insects have been presently recognized not only as a source of nutrition but also as a dietary component that possesses a broad range of activities against pathogens, attributed to their ability to produce antimicrobial peptides (AMPs). In addition, given that the nutrient composition of insects is influenced by their diet, it is possible that the nutritional value of insects could be transferred to animals when used as a feed source (Andreadis et al. 2021; Rumbos et al. 2022; Antonopoulou et al. 2022). Furthermore, it appears that the majority of the population is comfortable with consuming meat products derived from livestock that were fed with insect-based feed (Verbeke et al. 2015; Mancuso et al. 2016; Sebatta et al. 2018; Popoff et al. 2017; Ferrer Llagostera et al. 2019; Kulma et al. 2020; Rumbos et al. 2021a).

Insect production costs are multifaceted, with feedstock expenses being a significant contributor to the overall cost structure (Roffeis et al. 2018; Arru et al. 2019; Cadinu et al. 2020). To address this challenge, the utilization of low or economically insignificant substrates for insect feed has emerged as a viable strategy, capable of alleviating production expenses and subsequently reducing the market price of insect meal (Varelas 2019; Gasco et al. 2020). Given the substantial quantities of agricultural waste and by-products generated by farming and agroindustrial systems, these resources remain largely untapped as valuable substrates for insect rearing (FAO 2017). This approach aligns seamlessly with circular economy principles promoted within the European Union, bolstering the sustainability of insect farming. Recent research endeavors have explored the potential of utilizing agricultural post distillation residuals, demonstrating positive results (Kim et al. 2017; Mancini et al. 2019; Stull et al. 2019). Multiple studies have investigated the feasibility of valorizing such resources for various insect species, including the black soldier fly, *Hermetia illucens* (L.) (Diptera: Stratiomyidae) (Singh and Kumari 2019; Bosch et al. 2017); the long-horned grasshopper, *Ruspolia differens* (Serville) (Orthoptera: Tettigoniidae) (Sorjonen et al. 2020); the lesser mealworm, *Alphitobius diaperinus* (Panzer) (Gianotten et al. 2020; Van Broekhoven et al. 2015); and the yellow mealworm, *Tenebrio molitor* L*.* (Coleoptera: Tenebrionidae) (Oonincx et al. 2015; Van Broekhoven et al. 2015). Nevertheless, relative data related with the superworm, *Zophobas morio* (F.) (Coleoptera: Tenebrionidae), are limited (Rumbos and Athanassiou 2021a).

In the current study, we evaluated the performance of *Z. morio* larvae on diets enriched with functional ingredients of aromatic and medicinal plants of the Greek flora residues. *Zophobas morio* is an insect species that has been largely disregarded by both researchers and insect producers despite its considerable potential as a source of food and feed (Benzertiha et al. 2020; Rumbos and Athanassiou 2021a; Vasilopoulos et al. 2024). This neotropical beetle, classified as a member of the darkling beetles family Tenebrionidae, is widely utilized as a prevalent feed source for various animals, including birds, reptiles, and fish, due to its suitability and availability (Rumbos and Athanassiou 2021a). Recently, there has been a growing interest in utilizing organic by-products for *Z. morio* rearing purposes (Van Broekhoven et al. 2015; Harsányi et al. 2020). Larvae are commonly reared on wheat bran alone or augmented with a diverse range of cereal grains, such as oats, as well as other related amylaceous commodities. This versatility in feed options allows for flexible and customizable larval production methods (Quennedey et al. 1995; Aribi et al. 1997; Maciel-Vergara et al. 2018). Moreover, investigations into the nutritional composition of *Z. morio* larvae have consistently revealed their exceptional nutritional value, confirming their potential as a sustainable and nutrient-rich food source (Finke 2002, 2007, 2015; Ramos-Elorduy 2009; Barroso et al. 2014; Bosch et al. 2014; Adámková et al. 2016, 2017; Araújo et al. 2019).

The present study focuses on evaluating the performance of *Z. morio* larvae using diets enriched with functional ingredients derived from aromatic and medicinal plants of the Greek flora. Furthermore, the exceptional nutritional value of *Z. morio* larvae, as consistently demonstrated in previous studies, can be further improved by incorporating functional ingredients into their substrate, reinforcing their potential as a sustainable and highly nutritious food source (Finke 2002, 2007, 2015; Barroso et al. 2014; Bosch et al. 2014; Adámková et al. 2016, 2017; Araújo et al. 2019). Overall, this research contributes to the ongoing efforts to identify innovative and sustainable solutions to meet the growing global demand for food and feed resources.

## Materials and methods

### Insects

The culture of *Z. morio* used in this series of bioassays was maintained in the Laboratory of Entomology an Agricultural Zoology of the University of Thessaly (LEAZ, Volos, Magnesia, Greece) since 2018. The rearing conditions were 26 °C, 55% relative humidity (r.h.), and continuous darkness. Wheat bran was used as a rearing substrate, whereas insects were provided with agar cubes as a moisture source, three times a week.

Egg acquisition was performed by placing approximately 200 adults of *Z. morio* of mixed sex, in plastic containers (48-cm length × 28-cm width × 10-cm height) with flour, together with tangential cylindrical cartons. Female adults were left to lay their eggs on the contact points of the cartons for a week. After the termination of this interval, all adults were removed, and the eggs laid during that period were retained in the containers to allow hatching.

### Aromatic mixture

Two mixtures (A and B) of aromatic plants were tested. Mixture A was composed of post-distillation residues of essential oil extraction, obtained from various native aromatic plants found in Greece, including Crithmum plant residue (*Crithmum maritimum* L., oregano (*Origanum vulgare* subsp. *hirtum* (Link) Ietswaart), industrial cannabis (*Cannabis sativa* L.), linseed (*Linum usitatissimum* L.), and olive paste (*Olea europaea* L.) by-products (Table 1). Mixture B, on the other hand, was composed by the aforementioned ingredients (Table 1) supplemented with the essential oils presented in Table 2. The choice of these plants was guided by their local availability, given that it has been reported as one of the most crucial attributes for utilization in insect rearing (Rumbos et al. 2022).

**Table 1:** Composition of residue blend used as a substrate for insect feeding

	Mixture of plants and their residues	Concentration in aromatic mixture %
	Oregano residue	45
	Linseed	17
	Cannabis residue	17
	Olive paste	17
	Crithmum plant residue	4
	Total concentration	100

**Table 2:** Composition of essential blend used in Mixture B

	Mixture of essential oils	Concentration in insect feed g/kg
	Oregano essential oil	0.004
	Thyme essential oil	0.004
	Sage essential oil	0.004
	Rosemary essential oil	0.004
	Crithmum oil	0.004
	**Total**	**0.020**

### Bioassay I: evaluation of Mixture A

Wheat bran was supplemented with varying concentrations of Mixture A (10, 20, and 30%). Unenriched wheat bran was used as a control feed. All diets, before use, were air dried and pulverized in a mill (Ceccato M3, Ceccato Olindo, San Giorgio delle Pertiche PD, Italy). Plastic cylindrical vials, 7.5 cm in diameter and 8.8 cm in height, were used to contain 4 g of each substrate, with separate vials assigned to each substrate. Newly hatched larvae from the colony, aged < 3 days old, grouped in sets of 20 individuals, were weighed and transferred to the respective vials. In order to separate the larvae from the flour we used a 250-μm mesh sieve (Woven Wire Sieve, Endecotts Ldt, London, England). Afterwards, the larvae were allowed to feed without disturbance, over a period of 4 weeks. Carrot slices were provided as a moisture source, given to the larvae three times per week. After the 4-week period, the larvae were separated from the feeding substrate, and the survival rate and group larval weight were recorded. This process was reiterated biweekly until a minimum of 50% of the remaining larvae attained or surpassed a body length of 5 cm, reaching the final larval stage (van Broekhoven et al. 2015). This was done separately for each vial. In addition, the vials underwent visual inspection three times a week to check for any depletion of feed. In case the feed was found to be depleted, additional feed was introduced, and its weight was recorded. There were six replicates per dietary substrate.

### Bioassay II: evaluation of Mixture B

In this bioassay, we used *Z. morio* larvae <7 days old. To separate the larvae based on size, we used a 450-μm mesh sieve (Woven Wire Sieve, Endecotts Ldt, London, England), to retain only the larger larvae while allowing the smaller ones to pass through. We followed the same experimental procedure as previously described, with one key difference: In this case, we incorporated Mixture B instead of Mixture A in the aforementioned percentage levels.

### Calculations and statistical analysis

The development time was measured as the duration from the beginning of the experiment until at least 50% of the remaining larvae attained or surpassed a body length of 5 cm, reaching the final larval stage, within a vial. For the calculation of feed intake and conversion, it was assumed that all provided feed was consumed, while the weight of carrots was not considered in the calculations. In addition, we calculated feed utilization parameters, i.e., feed conversion ratio (FCR) and specific growth rate (SGR), using the following equations, as described by Rumbos et al. (2022), based on an earlier work of Waldbauer (1968):

1 $$\mathrm{Feed\;conversion\;ratio\;}({\text{FCR}})= \frac{\mathrm{Feed\;ingested}}{\mathrm{Live\;weight\;gained}}$$

2 $$\mathrm{Specific\;growth\;rate\;}\left({\text{SGR}}\right)\mathrm{\%}/{\text{day}}= 100\mathrm{\;x\;}\frac{\left(\mathrm{ln Final\;Bod\; weight}-\mathrm{lnInitial\;body\;weight}\right)}{{\text{days}}}$$

For each bioassay, all data were subjected to a one-way analysis of variance (ANOVA). Means were separated by Tukey HSD test at α 0.05. In this analysis, FCR, SGR, final survival rate, and final individual larval weight were considered as response variables, while the main factor under investigation was the rearing substrate. Before conducting the analysis, the data underwent tests to check for homogeneity of variance (Levene’s test) and normal distribution (Shapiro–Wilk test). For all cases, we used JMP 7 software (SAS Institute Inc., Cary, NC). All figures were created using Sigma-Plot software (Version 11, Systat, San Jose, CA, USA).

## Results

### Bioassay I: evaluation of Mixture A

**Fig. 1.**
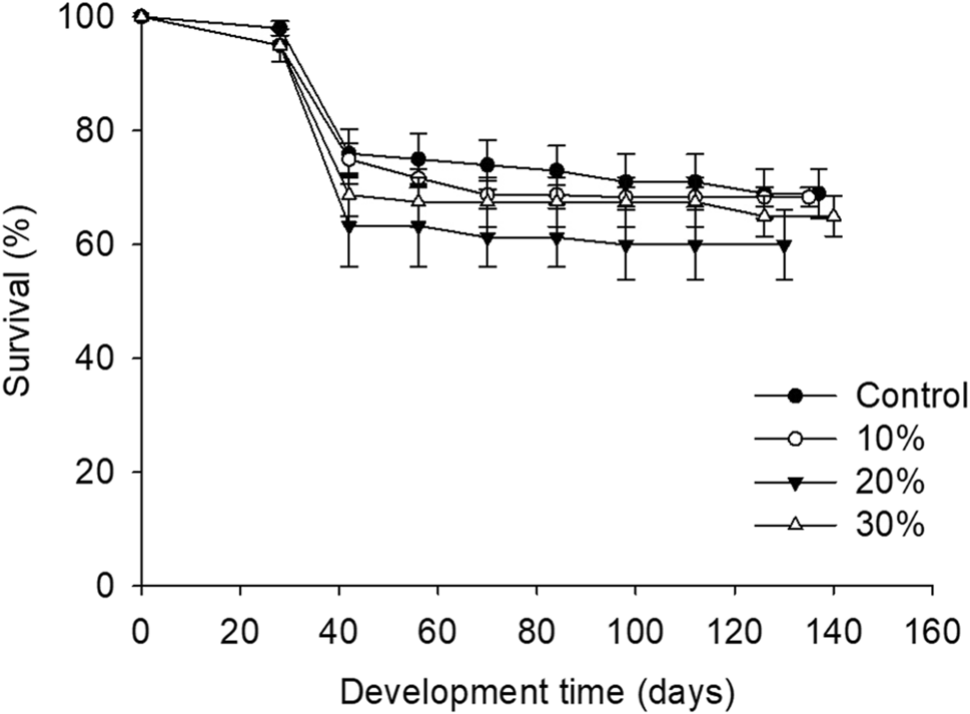
Survival rate (%) of *Zophobas morio* larvae reared on diets of wheat bran enriched with 0 (control), 10, 20, and 30% of Mixture A. For all treatments, values refer to mean ± SEM (*n* = 6)

The survival of *Z. morio* larvae on the tested diets and the control treatment is presented in Fig. 1. The lowest final average larval survival was recorded for larvae reared on 20% supplemented substrate and the highest on the control treatment; however. no significant difference was noted (Table 3).

**Table 3:** Final survival rate (%), final individual larval weight (mg), FCR, and SGR (%/day) of *Zophobas morio* larvae reared on diets of wheat bran enriched with 0 (control), 10, 20, and 30% of Mixture Α

Level of enrichment (%)	Survival (%)	Final individual larval weight (mg)	FCR	SGR (%/day)
0 (control)	69.0 ± 4.3	604.8 ± 26.1	1.9 ± 0.1	4.6 ± 0.1
10	68.3 ± 1.7	637.3 ± 7.7	1.9 ± 0.1	4.7 ± 0.1
20	60.0 ± 6.1	667.8 ± 14.6	2.0 ± 0.1	4.8 ± 0.1
30	65.0 ± 3.5	612.9 ± 23.2	1.9 ± 0.0	4.6 ± 0.1
F	0.50	1.94	0.13	0.43
*P*	0.84	0.16	0.93	0.98

Values represent means ± SEM (*n* = 6). Within each column, means followed by the same lowercase letter do not significantly differ according to Tukey HSD test (*p* < 0.05). Where no letters exist, no significant differences were noted. In all cases, df = 4.19

**Fig. 2.**
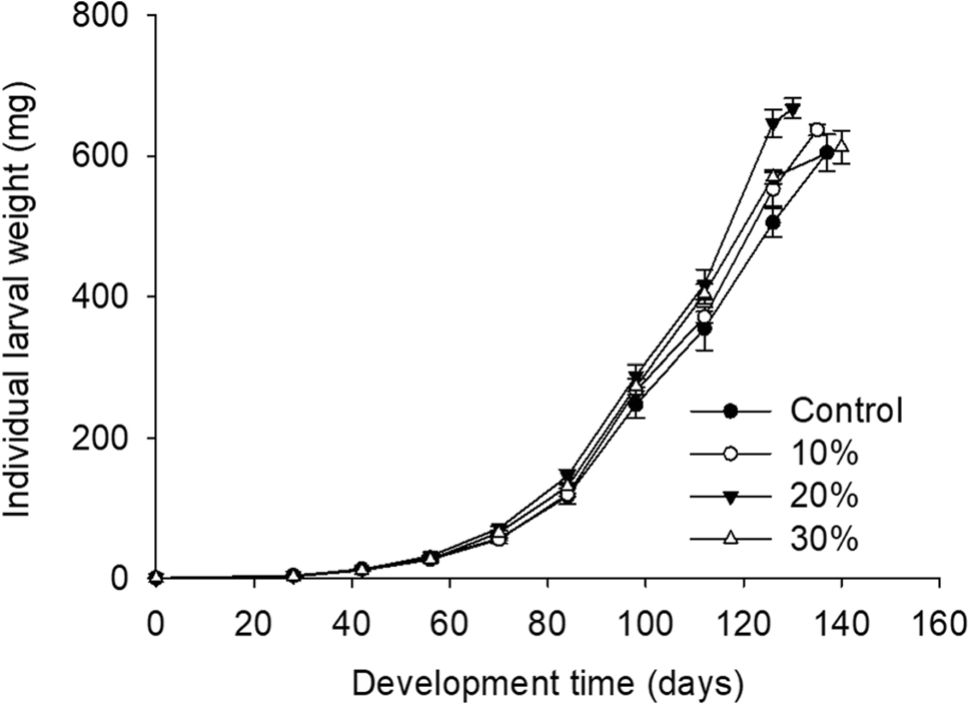
Individual larval weight (mg) of *Zophobas morio* larvae reared on diets of wheat bran enriched with 0 (control), 10, 20, and 30% of Mixture A. For all treatments, values refer to mean ± SEM (*n* = 6)

The weight gains of Z. *morio* larvae provided with diets supplemented with Mixture A were similar to larvae on the wheat bran control (Fig. 2). More specifically, the lowest final larval weight recorded was for larvae fed with the control substrate, and the highest was for larvae reared on 20% supplemented substrate; however, no significant difference was noted (Fig. 1; Table 3).

**Fig. 3.**
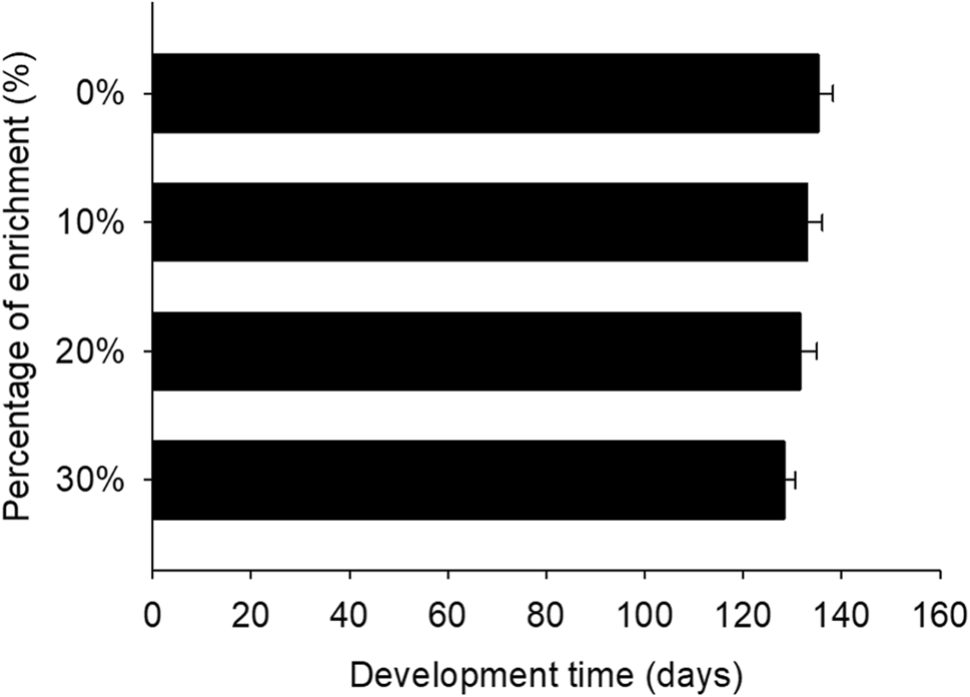
Development time (days) of *Zophobas morio* larvae reared on diets of wheat bran enriched with 0 (control), 10, 20, and 30% of Mixture A. For all treatments, values refer to mean ± SEM (*n* = 6)

Development time was not found to be significantly different among the different larval groups of each treatment. Hence, a 20% enrichment led to the shortest development time (130.7 days), while a 30% enrichment resulted in the longest (140.0) (df = 4.19; *F* = 4.00; *P* = 0.02) (Fig. 3). Regarding the feed utilization parameters, no significant differences were observed in FCR and SGR among larvae reared on the different diets (Table 3).

### Bioassay II: evaluation of Mixture B

**Fig. 4.**
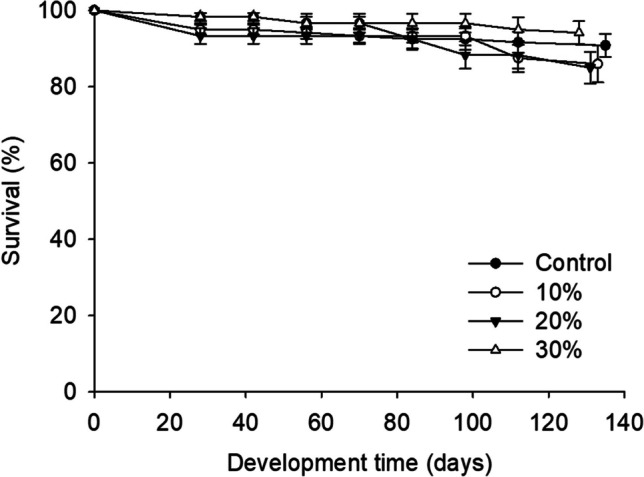
Survival rate (%) of *Zophobas morio* larvae reared on diets of wheat bran enriched with 0 (control), 10, 20, and 30% of Mixture B. For all treatments, values refer to mean ± SEM (*n* = 6)

**Fig. 5.**
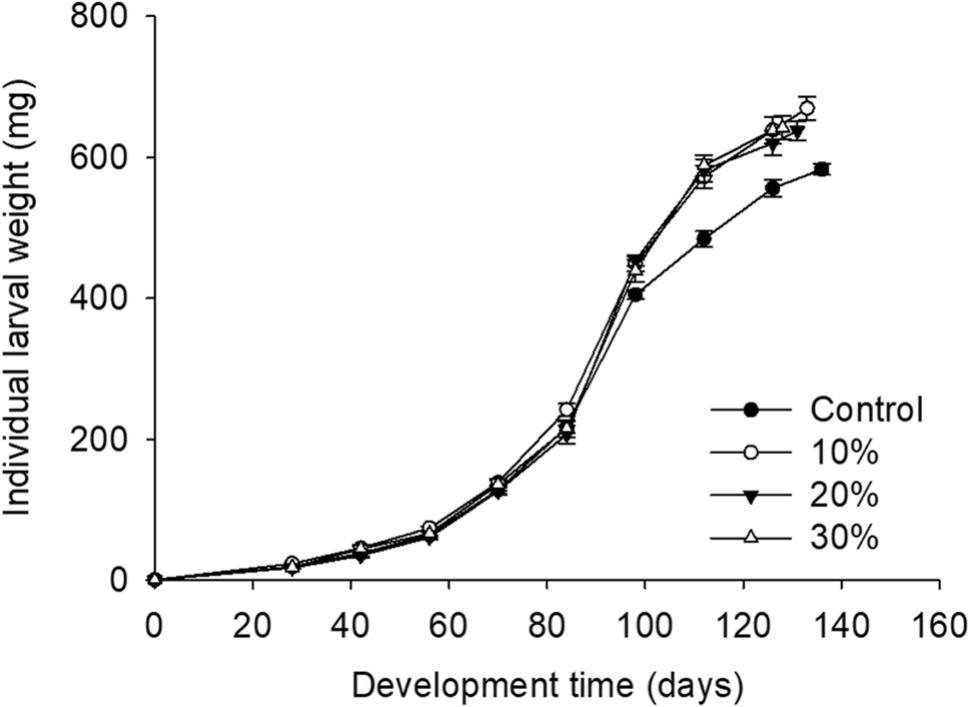
Individual larval weight (mg) of *Zophobas morio* larvae reared on diets of wheat bran enriched with 0 (control), 10, 20, and 30% of Mixture B. For all treatments, values refer to mean ± SEM (*n* = 6)

**Fig. 6.**
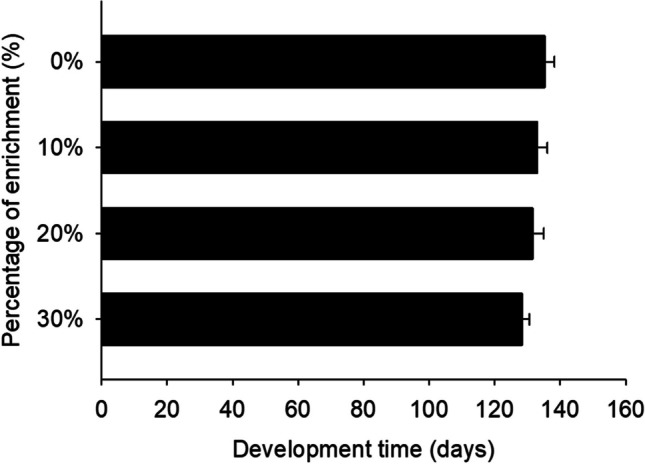
Development time (days) of *Zophobas morio* larvae reared on diets of wheat bran enriched with 0 (control), 10, 20, and 30% of Mixture B. For all treatments, values refer to mean ± SEM. Means followed by the same lowercase letter do not significantly differ according to Tukey HSD test (*p* < 0.05)

The final survival rates ranged from 84.0% for larvae reared on 20% supplemented substrate with Mixture B substrate to 94.2% for larvae reared on 30% (Fig. 4; Table 4). Figure 5 displays the weight gains observed in *Z. morio* larvae. The highest final larval weight of 669.5 ± 16.5 mg was recorded for the group of larvae reared at 30% supplemented substrate, while the control group exhibited the lowest average larval weight of 582.9 ± 7.8 mg. In addition, regarding development time, it was observed that larvae reared on 30% supplemented substrate exhibited the shortest development time (128.3 days), while those reared on the control diet experienced a significant longer development period (135.3) (df = 4.19; *F*= 2.4; *P* = 0.09) (Fig. 6). In terms of mean FCR, no significant differences were observed among the treatments (Table 4). However, the SGR varied significantly across the different diets. Notably, larvae reared with 30% supplemented substrate showed the highest SGR (5.1 ± 0.1 %/day), while the control group displayed the lowest (4.6 ± 0.1).

**Table 4:** Final survival rate (%), final individual larval weight (mg), FCR, and SGR (%/day) of *Zophobas morio* larvae reared on diets of wheat bran enriched with 0 (control), 10, 20, and 30% of Mixture B

Level of enrichment (%)	Survival (%)	Final individual larval weight (mg)	FCR	SGR (%/day)
0 (control)	89.2 ± 3.0	582.9 ± 7.8b	1.8 ± 0.1	4.6 ± 0.1b
10	82.0 ± 5.6	669.5 ± 16.5a	1.6 ± 0.1	5.0 ± 0.1ab
20	84.0 ± 4.6	637.8 ± 14.1ab	1.6 ± 0.1	5.0 ± 0.1ab
30	94.2 ± 3.0	642.1 ± 16.3a	1.5 ± 0.0	5.1 ± 0.1a
F	1.8	6.95	2.53	4.78
*P*	0.17	<0.001	0.10	0.015

Values represent means ± SEM (*n* = 6). Within each column, means followed by the same lowercase letter do not significantly differ according to Tukey HSD test (*p* < 0.05). Where no letters exist, no significant differences were noted. In all cases, df = 4.19

## Discussion

In the present study, we evaluated the larval development of *Z. morio*, on diets enriched with functional ingredients derived from aromatic and medicinal plants of the Greek flora. To our knowledge, this research represents the first investigation examining the impact of aromatic plants post-distillation residues and essential oil supplementation on the larval development of *Z. morio*. The larval development of insects is influenced by various factors, including the quality and composition of their diet (Rumbos et al. 2021b; Rumbos and Athanassiou 2021b; Gourgouta et al. 2022; Rumbos et al. 2022; Kotsou et al. 2021). The enrichment of diets with functional ingredients is a promising approach for enhancing the nutritional quality of insect diets, which can have implications for their growth and development but also affects their nutritional profile (Andreadis et al. 2021; Antonopoulou et al. 2022). Our findings indicate that the enrichment with the ingredients of both mixtures can positively affect larval development, as demonstrated by the survival, final larval weight, and development time of the larvae reared on the enriched diets.

Several studies have provided information on the dietary needs of *Z. morio*. These studies indicate that the larvae can be successfully reared on either wheat bran alone or with the addition of various cereal grains, such as oats and other starchy substances along with a moisture source (Quennedey et al. 1995; Aribi et al. 1997; Maciel-Vergara et al. 2018). In the case of our study, we used wheat bran as the main component of the diet. However, we also examined the impact of supplementing this base with two different mixtures at three varying levels of supplementation, with the upper limit set at 30%, and we did not go beyond this level of supplementation. This led to the creation of several distinct dietary formulations. The motivation behind this approach lies in the findings of numerous prior studies that have consistently reported the insecticidal effects of aromatic plants and essential oils on insects (Prakash et al. 2008; Weaver and Subramanyam 2000; Lampiri et al. 2020, Ayvaz et al. 2010). For instance, in a study conducted by Ayvaz et al. in 2010, the essential oils of oregano and savory (*Satureja hortensis* L.) exhibited remarkable efficacy against *Plodia interpunctella* (Hübner) and *Ephestia kuehniella* Zeller (Lepidoptera: Pyralidae), as the mortality rate reached 100% within 24 h at air concentrations of 9 µl/l for *P. interpunctella* and 25 µl/l for E. *kuehniella.* To the best of our knowledge, there is a lack of research investigating the insecticidal effect of botanical orientation oils on *Z. morio*. Interestingly, we observed that neither Mixture A nor Mixture B negatively affected the survival of *Z. morio* larvae. Regarding larvae reared in diets enriched with Mixture A, the larval survival rate, the final individual larval weight, and the development time remained unaffected for all levels of supplementation, resembling those of the control diet. On the other hand, supplementation with Mixture B, which included essential oils, demonstrated a positive impact on individual larval weight; however, no significant differences were observed among the different levels of implementation. The observed disparities in outcomes between larvae reared in diets enriched with Mixture A and Mixture B may be rooted in the diverse bioactive compounds present in each mixture, and potential complex interactions.

Unexpectedly, while supplementation with up to 20% of Mixture B had a positive effect on the development time of *Z. morio* larvae, higher concentration (30%) led to longer development time. This implies that elevated concentrations might have a more significant adverse effect on larval development. Similar results have been reported regarding *T. molitor* from a recent study by Andreadis et al. (2021). In that study, the authors examined the impact of various alternative diets, namely, rice bran, corncob, potato peels, solid biogas residues, and olive-oil processing residuals, in comparison to wheat bran (used as the control), on the growth and nutritional value of *T. molitor* larvae, in conjunction with the effect of post-distillation residues of Mediterranean aromatic-medicinal plants, i.e., lavender, Greek oregano, rosemary, and olive, in a 1:1:1:1 ratio when added at different levels (0, 10, and 20%) to the diets (Andreadis et al. 2021). The incorporation of 10% Mediterranean aromatic-medicinal plants increased total larval weights in rice bran, olive-oil processing residuals, solid biogas residues, and potato peels. However, further increase of Mediterranean aromatic-medicinal plants mixture to 20% did not lead to a significant alteration in the total larval weight. These findings are encouraging and highlight the potential of utilizing post distillation residuals and various categories of food waste, as dietary supplements for insect rearing.

Utilization of different types of byproducts has been previously investigated for the rearing of *Z. morio* larvae (Van Broekhoven et al. 2015; Nascimento et al. 2022). In a recent study, Nascimento et al. (2022) assessed the potential of utilizing grape residues for rearing and enhancing the nutritional value of *Z. morio* larvae. According to their findings, substituting 25% of the control diet (ground maize) with grape residue yielded results similar results to the control and proved more effective than replacements of 50%, 75%, and 100%. This is consistent with our findings, suggesting that using low levels of agricultural byproducts has no significant impact on larval performance, while higher levels may cause noticeable adverse effects in larval survival and development.

Van Broekhoven et al. (2015) evaluated the growth performance and FCR of three edible mealworm species, including *Z. morio*, on diets composed of organic by-products of spent grains and beer yeast, bread remains, cookie remains, potato steam peelings, and maize distillers’ dried grains (DDGS). According to the results of that work, while the dietary protein had a minor impact on the mealworms' protein content, its broader influence on overall development and survival is noteworthy. This is evident in the observation that the optimal FCR value of 2.7 was achieved with a high-protein, low-starch diet composed of beer yeast (40%), spent grains (30%), maize DDGS (20%), and bread remains (10%). The FCR values observed in our experiment were lower than those in the aforementioned study and ranged from 1.5 to 2.0. Higher FCR has also been observed for larvae reared on ground maize and a series of other substrates (Nascimento et al. 2022). These findings indicate that wheat bran, the main component in all tested diets, proved to be a favorable substrate for successful *Z. morio* larval rearing. Based on the above, the FCR of *Z. morio* larvae might be affected by the type of substrate and the composition of the diet, but this effect was not found to be highly detrimental in the treatments that were tested here.

In summary, our study demonstrates that the larval development of *Z. morio* is not negatively affected by the enrichment of diets with functional ingredients derived from aromatic and medicinal plants of the Greek flora. These findings highlight the potential of utilizing such ingredients to enhance the nutritional value of insect diets, with implications for animal feed production and sustainable food systems. Further research is warranted to explore the specific mechanisms and long-term effects of incorporating these functional ingredients into insect rearing practices.

### Supplementary Information

Below is the link to the electronic supplementary material.

Supplementary file1 (DOCX 23 KB)

## Data Availability

Data will be made available on request.

## References

[CR1] Adámková A, Kouřimská L, Borkovcová M, Kulma M, Mlček, J (2016) Nutritional values of edible Coleoptera (Tenebrio molitor, Zophobas morio and Alphitobius diaperinus) reared in the Czech Republic. Potravinarstvo 10:663–671

[CR2] Adámková A, Mlček J, Kouřimská L, Borkovcová M, Bušina T, Adámek M, Bednářová M, Krajsa J (2017) Nutritional potential of selected insect species reared on the island of Sumatra. J Environ Res Public Health 14:521 10.3390/ijerph14050521 PMC5451972 28498340

[CR3] Andreadis SS, Panteli N, Mastoraki M, Rizou E, Stefanou V, Tzentilasvili S, Sarrou E, Chatzifotis S, Krigas N, Antonopoulou E (2021). Towards functional insect feeds: agri-food by-products enriched with post-distillation residues of medicinal aromatic plants in *Tenebrio molitor* (Coleoptera: Tenebrionidae) breeding. Antioxidants.

[CR4] Antonopoulou E, Panteli N, Feidantsis K, Mastoraki M, Koutsogeorgiou E, Grivaki E, Papagrigoriou T, Christias S, Chatzifotis S, Lazari D, Andreadis S, Krigas N (2022). Carob (Ceratonia siliqua) as functional feed is beneficial in yellow mealworm (Tenebrio molitor) rearing: evidence from growth, antioxidant status and cellular responses. Antioxidants.

[CR5] Araújo RRS, dos Santos Benfica TAR, Ferraz VP, Moreira Santos E (2019). Nutritional composition of insects Gryllus assimilis and Zophobas morio: Potential foods harvested in Brazil. J Food Composition Anal.

[CR6] Aribi N, Quennedey A, Pitoizet N, Delbecque J-P (1997). Ecdysteroid titres in a tenebrionid beetle, Zophobas atratus: effects of grouping and isolation. J Insect Physiol.

[CR7] Arru B, Furesi R, Gasco L, Madau F, Pulina P (2019). The introduction of insect meal into fish diet: the first economic analysis on European sea bass farming. Sustainability.

[CR8] Ayvaz A, Sagdic O, Karaborklu S, Ozturk I (2010). Insecticidal activity of the essential oils from different plants against three stored-product insects. Journal of Insect Science.

[CR9] Barroso FG, de Haro C, Sánchez-Muros M-J, Venegas E, Martínez-Sánchez A, Pérez-Bañón C (2014). The potential of various insect species for use as food for fish. Aquaculture.

[CR10] Benzertiha A, Kierończyk B, Kołodziejski P, Pruszyńska-Oszmałek E, Rawski M, Józefiak D, Józefiak A (2020). Tenebrio molitor and Zophobas morio full-fat meals as functional feed additives affect broiler chickens’ growth performance and immune system traits. Poult Sci.

[CR11] Boland MJ, Rae AN, Vereijken JM, Meuwissen MPM, Fischer ARH, van Boekel MAJS, Rutherfurd SM, Gruppen H, Moughan PJ, Hendriks WH (2013). The future supply of animal-derived protein for human consumption. Trends Food Sci Technol.

[CR12] Bosch G, Zhang S, Oonincx DG, Hendriks WH (2014). Protein quality of insects as potential ingredients for dog and cat foods. J. Nutr. Sci..

[CR13] Bosch G, Fels-Klerx H, Rijk T, Oonincx D (2017). Aflatoxin B1 tolerance and accumulation in black soldier fly larvae (Hermetia illucens) and yellow mealworms (Tenebrio molitor). Toxins (Basel).

[CR14] Cadinu LA, Barra P, Torre F, Delogu F, Madau FA (2020). Insect rearing: potential, challenges, and circularity. Sustainability.

[CR15] FAO. 2019. The State of Food and Agriculture 2019. Moving forward on food loss and waste reduction. FAO, Rome, Italy

[CR16] FAO, WHO.  (2017). The State of Food and Agriculture: leveraging food systems for inclusive rural transformation.

[CR17] Ferrer Llagostera P, Kallas Z, Reig L, Amores de Gea D (2019). The use of insect meal as a sustainable feeding alternative in aquaculture: current situation, Spanish consumers’ perceptions and willingness to pay. J Clean Prod.

[CR18] Finke MD (2002). Complete nutrient composition of commercially raised invertebrates used as food for insectivores. Zoo Biol.

[CR19] Finke MD (2007). Estimate of chitin in raw whole insects. Zoo Biol.

[CR20] Finke MD (2015). Complete nutrient content of four species of commercially available feeder insects fed enhanced diets during growth. Zoo Biol.

[CR21] Gasco L, Biancarosa I, Liland NS (2020). From waste to feed: A review of recent knowledge on insects as producers of protein and fat for animal feeds. Curr Opin Green Sustain Chem.

[CR22] Gianotten N, Soetemans L, Bastiaens L (2020). Agri-food side-stream inclusions in the diet of Alphitobius diaperinus part 1: impact on larvae growth performance parameters. Insects.

[CR23] Gourgouta M, Rumbos CI, Michail V, Athanassiou CG (2022). Valorization of agricultural side-streams for the rearing of larvae of the lesser mealworm, Alphitobius diaperinus (Panzer). Sustainability.

[CR24] Harsányi E, Juhász C, Kovács E, Huzsvai L, Pintér R, Fekete G, Varga ZI, Aleksza L, Gyuricza C (2020). Evaluation of organic wastes as substrates for rearing Zophobas morio, Tenebrio molitor, and Acheta domesticus larvae as alternative feed supplements. Insects.

[CR25] Henry M, Gasco L, Piccolo G, Fountoulaki E (2015). Review on the use of insects in the diet of farmed fish: Past and future. Anim Feed Sci Technol.

[CR26] Kim SY, Kim HG, Yoon HJ, Lee KY, Kim NJ (2017). Nutritional analysis of alternative feed ingredients and their effects on the larval growth of Tenebrio molitor (Coleoptera: Tenebrionidae). Entomol Res.

[CR27] Kotsou K, Rumbos CI, Baliota GV, Gourgouta M, Athanassiou CG (2021). Influence of temperature, relative humidity and protein content on the growth and development of larvae of the lesser mealworm, Alphitobius diaperinus (Panzer). Sustainability.

[CR28] Koutsos L, McComb A, Finke M (2019). Insect composition and uses in animal feeding applications: a brief review. Ann Entomol Soc Am.

[CR29] Kulma M, Kouřimská L, Homolková D, Božik M, Plachý V, Vrabec V (2020). Effect of developmental stage on the nutritional value of edible insects A case study with Blaberus craniifer and Zophobas morio. J Food Comp Anal.

[CR30] Lampiri E, Agrafioti P, Levizou E, Athanassiou CG (2020). Insecticidal effect of Dittrichia viscosa lyophilized epicuticular material against four major stored-product beetle species on wheat. Crop Protection.

[CR31] Li L (2023). Commodity prices volatility and economic growth: empirical evidence from natural resources industries of China. Resources Policy.

[CR32] Maciel-Vergara G, Jensen A, Eilenberg J (2018). Cannibalism as a possible entry route for opportunistic pathogenic bacteria to insect hosts, exemplified by Pseudomonas aeruginosa, a pathogen of the giant mealworm Zophobas morio. Insects.

[CR33] Makkar HPS, Tran G, Heuzé V, Ankers P (2014). State-of-the-art on use of insects as animal feed. Anim Feed Sci Technol.

[CR34] Mancini S, Fratini F, Turchi B, Mattioli S, Dal Bosco A, Tuccinardi T, Nozic S, Paci G (2019). Former foodstuff products in Tenebrio molitor rearing: effects on growth, chemical composition, microbiological load, and antioxidant status. Animals.

[CR35] Mancuso T, Baldi L, Gasco L (2016). An empirical study on consumer acceptance of farmed fish fed on insect meals: the Italian case. Aquaculture Int.

[CR36] Msangi S, Rosegrant MW (2011) Feeding the future’s changing diets: implications for agriculture markets, nutrition, and policy. In 2020 Conference: Leveraging Agriculture for Improving Nutrition and Health. Washington, DC: Int Food Pol Res Inst

[CR37] Nascimento RQ, Di Mambro Ribeiro CV, Colauto NB, da Silva L, Lemos PVF, de Souza Ferreira E, Linde GA, Machado BAS, Tavares PPLG, Biasoto ACT, Umsza Guez MA, Carvalho N, de Jesus Assis D, da Silva JBA, de Souza CO (2022). Utilization of agro-industrial residues in the rearing and nutritional enrichment of Zophobas atratus larvae: new food raw materials. Molecules.

[CR38] Nelson GC, Rosegrant MW, Koo J, Robertson R, Sulser T, Zhu T., Ringler C, Msangi S, Palazzo A, Batka M, Magalhaes M, Valmonte-Santos R, Ewing M, Lee D (2009). Climate change: impact on agriculture and costs of adaptation (Vol. 21). Intl Food Policy Res Inst

[CR39] OECD-FAO Agricultural Outlook 2018–2027. OECD Publishing, Paris/Food and Agriculture Organization of the United Nations, Rome, Italy. 10.1787/agr_outlook-2018-en

[CR40] Oonincx DG, Van Broekhoven S, Van Huis A, van Loon JJ (2015). Feed conversion, survival and development, and composition of four insect species on diets composed of food by-products. PloS one.

[CR41] Popoff M, MacLeod M, Leschen W (2017). Attitudes towards the use of insect-derived materials in Scottish salmon feeds. J Insects Food Feed.

[CR42] Prakash A, Rao J, Nandagopal V (2008). Future of botanical pesticides in rice, wheat, pulses and vegetables pest management. J. Biopest..

[CR43] Quennedey A, Aribi N, Everaerts C, Delbecque J-P (1995). Postembryonic development of Zophobas atratus Fab. (Coleoptera: Tenebrionidae) under crowded or isolated conditions and effects of juvenile hormone analogue applications. J Insect Physiol.

[CR44] Ramos-Elorduy J (2009). Anthropo-entomophagy: cultures, evolution and sustainability. Entomol Res.

[CR45] Roffeis M, Wakefield ME, Almeida J, Alves Valada TR, Devic E, Koné N, Kenis M, Nacambo S, Fitches EC, Koko GKD, Mathijs E, Achten WMJ, Muys B (2018). Life cycle cost assessment of insect based feed production in West Africa. J Clean Prod.

[CR46] Rumbos CI, Bliamplias D, Gourgouta M, Michail V, Athanassiou CG (2021). Rearing Tenebrio molitor and Alphitobius diaperinus larvae on seed cleaning process byproducts. Insects.

[CR47] Rumbos CI, Mente E, Karapanagiotidis IT, Vlontzos G, Athanassiou CG (2021). Insect-based feed ingredients for aquaculture: a case study for their acceptance in Greece. Insects.

[CR48] Rumbos CI, Oonincx DGAB, Karapanagiotidis IT, Vrontaki M, Gourgouta M, Asimaki A, Mente E, Athanassiou CG (2022). Agricultural by-products from Greece as feed for yellow mealworm larvae: circular economy at a local level. J Insects Food Feed.

[CR49] Rumbos CI, Athanassiou CG (2021a) The Superworm, Zophobas morio (Coleoptera:Tenebrionidae): a ‘sleeping giant’ in nutrient sources. J Insect Sci 21. 10.1093/jisesa/ieab014 10.1093/jisesa/ieab014 PMC8033247 33834209

[CR50] Rumbos CI, Athanassiou CG (2021b) ‘Insects as food and feed: if you can’t beat them, eat them!’—to the magnificent seven and beyond. J Insect Sci. 21. 10.1093/jisesa/ieab019 10.1093/jisesa/ieab019 PMC8023366 33822126

[CR51] Searchinger T, Waite R, Hanson C, Ranganathan J, Dumas P, Matthews E (2018) Creating a sustainable food future—a menu of solutions to feed nearly 10 billion people by 2050. World Resources Institute, Washington, DC. Available at https://files.wri.org/s3fs-public/creating-sustainable-food-future_2.pdf

[CR52] Sebatta C, Ssepuuya G, Sikahwa E, Mugisha J, Diiro G, Sengendo M, Fuuna P, Fiaboe KKM, Nakimbugwe D (2018). Farmers’ acceptance of insects as an alternative protein source in poultry feeds. Int J Agric Res Innov Technol.

[CR53] Singh A, Kumari K (2019). An inclusive approach for organic waste treatment and valorisation using Black Soldier Fly larvae: a review. J Environ Manage.

[CR54] Sorjonen JM, Lehtovaara VJ, Immonen J, Karhapää M, Valtonen A, Roininen H (2020). Growth performance and feed conversion of Ruspolia differens on plant-based by-product diets. Entomol Exp Appl.

[CR55] Stull VJ, Kersten M, Bergmans RS, Patz JA, Paskewitz S (2019). Crude protein, amino acid, and iron content of Tenebrio molitor (Coleoptera, Tenebrionidae) reared on an agricultural byproduct from maize production: an exploratory study. Ann Entomol Soc Am.

[CR56] Teguia A, Beynen A (2005). Alternative feedstuffs for broilers in Cameroon. Livest. Res. Rural Dev..

[CR57] van Huis A, Gasco L (2023). Insects as feed for livestock production. Science.

[CR58] van Huis A, Oonincx DGAB (2017). The environmental sustainability of insects as food and feed. A review. Agron Sustain Dev.

[CR59] van Broekhoven S, Oonincx DGAB, van Huis A, van Loon JJA (2015). Growth performance and feed conversion efficiency of three edible mealworm species (Coleoptera: Tenebrionidae) on diets composed of organic by-products. J Insect Physiol.

[CR60] Varelas V (2019). Food wastes as a potential new source for edible insect mass production for food and feed: a review. Fermentation.

[CR61] Vasilopoulos S, Giannenas I, Panitsidis I, Athanassiou C, Papadopoulos E, Phortomaris P (2024). Effect of three different insect larvae on growth performance and antioxidant activity of thigh, breast, and liver tissues of chickens reared under mild heat stress. Trop Anim Health Prod.

[CR62] Veldkamp T, Van Duinkerken G, van Huis A, Lakemond CMM, Ottevanger E, Bosch G, Van Boekel T (2012). Insects as a sustainable feed ingredient in pig and poultry diets - A feasibility study. In: Report 638. Wageningen UR Livestock Research.

[CR63] Verbeke W, Spranghers T, De Clercq P, De Smet S, Sas B, Eeckhout M (2015). Insects in animal feed: acceptance and its determinants among farmers, agriculture sector stakeholders and citizens. Anim Feed Sci Technol.

[CR64] Weaver DK, Subramanyam, B (2000) Botanicals. Alternatives to pesticides in stored-product IPM 303–320

[CR65] Zhao L, Chau KY, Tran TK, Sadiq M, Xuyen NTM, Phan TTH (2022). Enhancing green economic recovery through green bonds financing and energy efficiency investments. Econ Anal Policy.

