# Supplementary material for: Larval performance of Zophobas morio (F.) (Coleoptera: Tenebrionidae) on various diets enriched with post-distillation residues and essential oils of aromatic and medicinal plants
Source: Environ Sci Pollut Res Int. 2024 Apr 2;31(20):28847–55. doi: 10.1007/s11356-024-32603-8 (PMC11058798; doi:10.1007/s11356-024-32603-8)
Supplement: Supplementary file 1 — Supplementary file1 (DOCX 23 KB) [file 11356_2024_32603_MOESM1_ESM.docx]

**Supplementary material**

**Analysis of essential oil mixture distilled from substrates by steam-distillation**

Gas chromatography-mass spectrometry (GC-MS) was used to determine the composition of the essential oil. The separation of compounds was performed on an HP-5MS column (Agilent, 30m x 0.25mm x 0.25μm). The analysis conditions were as follows: Injection point temperature: 260ºC, ionization source temperature 200ºC, GC-MS junction temperature: 300ºC, electron ionization type: 70 eV, scan range: 41 – 450 amu, scan time 0.50 s.

The following temperature programs were applied: a) 55 – 120ºC (3ºC/min), 120 – 200ºC (4ºC/min), 200 – 220ºC (6ºC/min) and 220ºC for 5 min; and b) 60 – 240ºC at a rate of 3ºC/min. Carrier Gas He, 54.8 kPa, input sample ratio 1:30. The relative content of each compound was calculated as a % of the total chromatogram and the results presented in Table 1 are the average of two technical replicates.

The identification of the components of the oils was based on the comparison of their Kovats indices in relation to those of known alkanes (standard n-alkanes) with corresponding data from the literature, as well as comparing: a) their spectra with corresponding MS mass spectra (Adams , NIST 98, Willey, 1995) and b) the retention time (RT) of each compound, with corresponding reference substances.

Table 1. Composition (%) of the essential oil mixture (oregano : thyme : sage : rosemary : kritam, 1:1:1:1:1).

| **Ingredients** | **Elution time** | **%** |
| --- | --- | --- |
| Tricyclene | 7,17 | 0,05 |
| α-Thujene | 7,374 | 0,64 |
| α-Pinene | 7,649 | 5,74 |
| Camphene | 8,254 | 2,08 |
| Verbenene | 8,511 | 0,07 |
| Sabinene | 9,425 | 2,97 |
| β-Pinene | 9,55 | 0,85 |
| 1-Octen-3-ol | 9,742 | 0,16 |
| 3-Octanone | 10,12 | 1,56 |
| β-Myrcene | 10,358 | 2,53 |
| 3-Octanol | 10,602 | 0,1 |
| a-Phellandrene | 11,001 | 0,63 |
| 3-d-Carene | 11,319 | 0,06 |
| α-terpinene | 11,699 | 1,8 |
| p-Cymene | 12,185 | 6,29 |
| Limonene+β-Phellandrene | 12,432 | 6,97 |
| 1,8-Cineole | 12,559 | 8,12 |
| cis-β-Ocimene | 13,079 | 0,79 |
| trans-β-ocimene | 13,716 | 0,08 |
| γ-Terpinene | 14,359 | 9,78 |
| trans- sabinene hydrate | 14,817 | 0,51 |
| α-terpinolene | 16,264 | 0,55 |
| cis-Sabinene hydrate | 16,933 | 0,21 |
| Linalool | 17,236 | 0,67 |
| cis-Thujone | 17,464 | 4,57 |
| trans-Thujone | 18,229 | 1,28 |
| Camphor | 20,173 | 5,16 |
| Borneol | 21,837 | 1,43 |
| 4-Terpineol | 22,8 | 1,37 |
| α-Terpineol | 23,902 | 0,58 |
| Verbenone | 25,5 | 0,28 |
| Thymol methyl ether | 27,649 | 0,65 |
| α-Fenchyl acetate | 31,818 | 0,6 |
| Thymol | 32,63 | 2,58 |
| Carvacrol | 33,563 | 22,05 |
| α-Copaene | 38,493 | 0,07 |
| β-Caryophyllene | 41,791 | 3,28 |
| α-Humulene | 43,884 | 0,64 |
| γ-Muurolene | 45,365 | 0,15 |
| α-Copaene | 46,323 | 0,09 |
| β-Bisabolene | 47,208 | 0,06 |
| D-Germacrene | 47,332 | 0,07 |
| δ-Cadinene | 47,877 | 0,24 |
| p-Cymene-2,5-diol | 49,637 | 0,14 |
| Caryophyllene oxide | 50,573 | 0,23 |
| Globulol | 50,988 | 0,39 |
| Dillapiole | 52,432 | 0,74 |
| Σύνολο ταυτοποιημένων ενώσεων αιθερίου ελαίου | | 99,86 |

According to the results presented in Table 1, 99.86% of the total essential oil was identified. The compounds of the essential oil blend belong to the following broad groups: hydrocarbon-monoterpenes, oxygenated monoterpenes, sesquiterpenes and benzodioxoles (with dillapiole as the main component). The main components of the mixture detected were carvacrol, γ-terpinene, para-cymene, 1,8-cineole, camphor, α-pinene, and β-phellandrene. At a relative concentration lower than 5% the following were detected: cis-thujone, camphene, thymol, β-myrcene, β-caryophyllene, savinene and α-terpinene. More specifically, the compounds γ-terpinene, para-cymene, carvacrol and thymol are characteristic components of the essential oil of oregano and thyme. At the same time, 1,8-cineol, camphor, cis-/trans-thujones are basic components of sage essential oils, while the first two compounds together with borneol and verbenone are usually contained in rosemary essential oils. Finally, Crithmum essential oil is represented by the presence of β-phellandrene and dill-apiol, which is a basic "bio-indicator" of this particular essential oil.

**Determination of antioxidants in the substrate (by-product of the distillation of essential oils)**

Extraction of antioxidants

A methanolic extract of the dried and ground substrate was used to determine the antioxidant capacity, water-soluble phenols and flavonoids. More specifically, 0.2 g of dried tissue was homogenized with 10 ml of 70% (v/v) methanol and incubated for 15 min in an ultrasonic bath. Then, they were centrifuged (at 4°C with a relative centrifugation force of 12,000 RCF for 20 minutes) and the supernatant extract was collected in a clean vial.

Quantification of total water-soluble phenols

The concentration of total water-soluble phenols in the methanol extracts of the substrate was determined using the Folin-Ciocalteu reagent as described by Scalbert et al. (1989), using gallic acid as a standard substance. Samples were measured in triplicate and sample values were expressed as mg gallic acid equivalents/g dry tissue.

Quantification of flavonoids

A modified method was used to determine the concentration of flavonoids as previously described by Zhishen et al. (1999). To determine the concentration of flavonoids, a reference curve was prepared using catechin as a standard substance. Samples were measured in triplicate and their values were expressed as mg catechin equivalents/g dry tissue.

Determination of antioxidant capacity

In order to determine the antioxidant capacity of the methanol extracts, the ABTS (Sarrou et al., 2016) and DPPH (Su et al., 2006) tests were used. These tests are quite fast, non-specific and direct spectrophotometric methods by which the total antioxidant capacity of a plant extract is calculated (Table 2).

|  |  |
| --- | --- |
| Total water-soluble phenols mg GA^1^/g ΞΒ) | 9,45 |
| Total flavonoids (mg CA^2^/g ΞΒ) | 6,96 |
| Antioxidant potential *ABTS* (mg Tr^3^/g ΞΒ) | 22,39 |
| Antioxidant potential *DPPH* (mg Tr/g ΞΒ) | 19,9 |

^1^GA: Gallic acid

^2^CA: catechin

^3^Tr: Trolox

References

Sarrou E, Martens S, Chatzopoulou P (2016). Metabolite profiling and antioxidative activity of Sage (*Salvia fruticosa* Mill.) under the influence of genotype and harvesting period.  Ind Crops Prod 94: 240-250.

Zhishen J, Mengcheng T, Jianming W (1999). The determination of flavonoid contents in mulberry and their scavenging effects on superoxide radicals. Food Chem , *64*: 555-559.

Su MS, Silva JL (2006). Antioxidant activity, anthocyanins, and phenolics of rabbiteye blueberry (Vaccinium ashei) by-products as affected by fermentation.  Food Chem 97: 447-451.
